# Effect of the Application of a Green Preservative Strategy on Minced Meat Products: Antimicrobial Efficacy of Olive Mill Wastewater Polyphenolic Extract in Improving Beef Burger Shelf-Life

**DOI:** 10.3390/foods11162447

**Published:** 2022-08-14

**Authors:** Rossana Roila, Beatrice Sordini, Sonia Esposto, David Ranucci, Sara Primavilla, Andrea Valiani, Agnese Taticchi, Raffaella Branciari, Maurizio Servili

**Affiliations:** 1 Department of Veterinary Medicine, University of Perugia, Via San Costanzo 4, 06126 Perugia, Italy; 2 Department of Agricultural, Food and Environmental Sciences, University of Perugia, 06126 Perugia, Italy; 3 Istituto Zooprofilattico Sperimentale dell’Umbria e delle Marche “Togo Rosati”, Via G. Salvemini 1, 06126 Perugia, Italy

**Keywords:** microbial spoilage, lipidic oxidation, antioxidant, predictive microbiology, food preservation, food safety, sustainable strategy, by-product reuse, kinetic parameters, *Olea europaea*

## Abstract

The mincing process of raw meat favors microbial spoilage as well as chemical and enzymatic oxidation processes. In order to limit this degradative process, preservatives are routinely added to minced meat products. The role of olive mill wastewater polyphenolic extract as a replacement for synthetic preservatives in beef burger was assessed. The antioxidant capacity of the extract experimentally added to beef burger was evaluated using the oxygen radical absorbance capacity method (ORAC_FL_) to assess the shelf-life, while the lipid oxidation was measured by thiobarbituric reactive substance (TBAR) determination. The antimicrobial activity was assayed by means of classical methods and predictive microbiology. The experimental addition of polyphenolic extract led to 62% lower lipid oxidation and 58% higher antioxidant capacity; it also successfully modulated spoilage microbial populations with an average growth reduction of 15% on day 7. Results indicate that olive mill wastewater polyphenolic extracts could be added to raw ground beef meat to act as natural antioxidants and to modulate microbial growth.

## 1. Introduction

Burger is one of the most appreciated meat products worldwide for its ease of preparation and versatility of consumption, which is considered a time-saving strategy in the modern lifestyle [1,2,3]. Nevertheless, the grinding process for raw meat, resulting in the disruption of muscle structure, leads to a less stable food matrix favoring microbial spoilage as well as chemical and enzymatic oxidation processes with possible repercussions on safety and health [4,5]. Several strategies, such as peculiar production processes, packaging and food additives, have been studied during the last few decades in order to reduce the above-mentioned phenomena and enhance the shelf-life of these meat products [6,7,8,9,10]. Studies in the literature demonstrate that antioxidant molecules protect the grinded meat from oxidation and delay the microbial growth [11]. Consequently, in fresh ground meat preparations, additives with antioxidant properties are usually employed. Although food additives are strictly regulated (Regulation (EC) No. 1333/2008, s.m.i.) [12], chemical compounds intentionally added into food are considered with mistrust by consumers due to their potential long-term adverse effects linked to hypersensitivity, asthma, cancer, skin irritation, allergies and gastrointestinal problems [13,14]. As a consequence, growing interest has been demonstrated by consumers towards products with natural antioxidants, encouraging food industries to continuously research for the newest natural food additives to replace synthetic ones [15].

Several spices, essential oils, extracts, powders and other plant by-products have been studied in recent decades in order to assess their activity and their effects on meat products as food supplementation [16,17,18,19]. Among these, olive oil by-products can be considered a source of bioactive molecules that are potentially applicable for processed meat preservation [20]. It is known that olive oil by-products are characterized by a high number of hydrophilic phenols, mainly secoiridoids, found exclusively in the *Oleaceae* family, that have been proven to inhibit or delay the rate of growth of a wide range of Gram-positive and Gram-negative bacteria as well as to have high antioxidant properties [21,22]. In particular, the olive mill wastewater generated in olive oil production has a high generation rate (49% of total mass), and the possible exploitation of this agro-industrial waste through the recovery of high-value bioactive compounds could positively affect the economic and environmental sustainability of agro-industry [23].

Although the chemical composition and the antioxidant capacity of olive oil by-products as well as their application in foodstuff have been previously studied by several authors [20,24,25], information on the effect of the olive mill wastewater polyphenolic extract on the microbial population of minced meat products to improve their shelf-life is still limited. 

In this sense, the role of this natural extract in the replacement of synthetic preservatives in meat products is postulated. With this aim, the antioxidant activity and antimicrobial capacity of olive mill wastewater polyphenolic extract during beef burger shelf-life was evaluated. In order to estimate the potential activity of this polyphenolic extract in comparison with synthetic additives, different formulations were tested and compared to a control formulation and a control formulation with synthetic preservatives (sodium ascorbate) during a period of 7 days of cold storage.

## 2. Materials and Methods

### 2.1. Olive Mill Waste Water Extract and Compositon

The crude phenolic extract (PE) used in the beef burger formulation was obtained through a three-step membrane filtration process using fresh olive mill wastewaters from processing olives of the Umbrian Moraiolo cultivar, as reported by Ianni et al. [26]. To obtain a stable powder formulation, the extracts were spray-dried after their combination with maltodextrin (1:1 dw), which functioned as a carrier, and is largely used in spray drying in the food industry [27]. The phenolic composition of the spray-dried extracts was assessed by means of high-performance liquid chromatography (HPLC) [28] and is reported in Table 1.

### 2.2. Beef Burger Formulation

Beef burgers were produced in an EU-approved meat processing plant located in Umbria, Central Italy. The meat for the preparation of beef burger was obtained from cuts (beef rump and shoulder muscle) of 18-month-old female Chianina cattle reared and slaughtered in Italy in accordance with European Union Regulation (Regulation (EC) No. 853/2004 s.m.i.) [29]. After 12 days of carcass aging, 40 kg of meat cuts was ground twice in a professional trimmer equipped with a 4 mm-hole plate. The grinded meat was divided into four different formulations, each 5 kg in weight, and aseptically hand-mixed with the following ingredients for 2 min (basic recipe): 10 g/kg NaCl, 60 g/kg grated parmesan, 80 g/kg breadcrumbs and 2 eggs/kg. The four different formulations were elaborated as follows: C, basic recipe with no addition; A, basic recipe plus 10 g/kg commercial antioxidant mix (CM) (CondiHamb, MEC Import, Perugia, Italy); AP, basic recipe plus 5 g/kg CM plus 350 mg/kg PE crude extract; P, basic recipe plus 700 mg/kg PE crude extract. The chemical composition of the burger was characterized by an average of 22% protein and 8 % fat. Each batch was further mixed for 2 min, and the burgers were then molded (about 100 g each) and placed in a display refrigerator at 4 ± 2 °C for 7 days, under alternating exposure to fluorescent light (12 h light/12 h darkness) to simulate retail storage conditions.

### 2.3. Antioxidant Capacity of PE and Mix Extracts and Beef Burger

The antioxidant capacity was evaluated for the synthetic and natural additives and burgers over the course of their shelf-life using the oxygen radical absorbance capacity method (ORAC_FL_). One gram each of CM, PE and burger samples was separately mixed with a buffer, 75 mM, pH 7.2, containing 13.19 g of K_2_HPO_4_ and 10.26 g of KH_2_PO_4_ in 900 mL of deionized water, homogenized with an Ultra-Turrax homogenizer (Ultra Turrax T25 Basic, IKA Labortechnik Janke & Kunkel GmbH, Stavfen, Germany) for 1 min, and then vortexed for 2 min. The homogenates were centrifuged at 6000 rpm at 4 °C for 20 min, and the supernatant was used for the determination of the antioxidant capacity using the oxygen radical absorbance capacity method (ORAC_FL_) based on the fluorescence decay rate of a probe in the presence of a radical oxygen species (ROO) and compared with that of a reference standard, Trolox (6-hydroxy-2,5,7,8-tetramethylchroman-2-carboxylic acid, Sigma-Aldrich, Steinheim, Germany). The ORAC_FL_ assays were carried out on a FLUO-star OPTIMA microplate fluorescence reader (BMGLABTECH, Offenburg, Germany) at an excitation wavelength of 485 nm and an emission wavelength of 520 nm. The results are expressed as µg of Trolox equivalents (TE) per 100 g sample.

### 2.4. Lipid Oxidation of Beef Burger

Lipid oxidation over the course of shelf-life was measured by thiobarbituric reactive substance (TBAR) determination, performed according to Tarladgis et al. [30] and measured by an Ultrospec 2100 pro UV–visible spectrometer (Amersham Pharmacia Biotech, Amersham, UK) at 532 nm. Quantification was performed using a standard calibration curve and with a concentration range of 1E6 to 1E5 M (y = 2E + 07x + 0.0046, R^2^ = 0.9999), corresponding to a range of 0.4–4 mg of malonaldehyde (MDA)/kg meat. The MDA recovery was determined by spiking the samples with a known volume of 0.2 mM TMP. The TBAR concentration was expressed as mg MDA/kg meat.

### 2.5. Antimicrobial Activity of Commercial Mix and PE Extract

The evaluation of the antimicrobial activity of CM and PE extract was performed through the agar well diffusion technique [31,32] on some micro-organisms relevant for the food industry, such as *Staphylococcus aureus* (WDCM 00034), *Escherichia coli* (WDCM 00013) and *Pseudomonas fluorescens* (WDCM 00115). The selected reference strains were revitalized in Brain Heart Infusion (BHI) broth and incubated at 37 °C for 24 h with the exception of *P. fluorescens*, which was incubated at 25 °C for 24–48 h. An initial suspension of 0.5 McFarland in 0.9% sterile saline solution was prepared for each micro-organism, and 100 µL was then distributed on Mueller–Hinton Agar (MHA, Thermo Fisher Scientific, Milan, Italy) plates with a swab, making four 90° rotations. At the time of use, the extracts were suspended with sterile demineralized water to obtain a concentration of 750 mg/mL, and then two dilutions were performed at 375 and 187 mg/mL. In each inoculated MHA plate, 7 mm-diameter holes were produced with a sterilized cork borer and then filled with 50 µL of extract suspension at different concentrations [31,32]. The plates were incubated according to the most suitable growth conditions, as reported above. For each bacterial strain, CM and PE extract were tested, and a negative control was set up with sterile demineralized water. At the end of the incubation period, the diameter of the inhibition halo was measured by a gauge and expressed in mm.

### 2.6. Microbial Analysis of Beef Burger

After 0, 2, 5 and 7 days of storage (T0, T1, T2 and T3, respectively), 10 g of each sample was aseptically removed and placed in a sterile stomacher bag with 90 mL of Buffered Peptone Water (Oxoid Ltd., Basingstoke, UK). After homogenization (Stomacher 400 circulator, Seward Ltd., Norfolk, UK), decimal serial dilutions were performed, and the below-reported microbiological determination was carried out in duplicate. Total viable count (TVC) was performed on Plate Count Agar (PCA, Oxoid Ltd.) incubated at 30 °C for 72 h according to ISO 4833-1 [33]. *Enterobacteriaceae* were enumerated according to a validated alternative method of ISO 21528-2 [34] (AFNOR AES 10/07-01/08) on Rebecca™ EB (bioMérieux, Mercy Etoile, France) incubated at 37 °C for 24 h. The lactic acid bacteria (LAB) count was performed on de Man, Rogosa and Sharpe agar (MRS, Oxoid Ltd.) incubated at 30 °C for 72 h, while the *Pseudomonas* spp. count was performed on Pseudomonas Agar Base with CFC selective agar supplement (Oxoid Ltd.) and incubated for 48 h at 25 °C. Coagulase-positive staphylococci were enumerated on Baird Parker Agar with the addition of RPF supplement (Biolife, Milano, Italy) and incubated at 37 °C for 48 h. Results were recorded as colony-forming units (CFUs) and converted into log10 values to obtain Log CFU/g of meat prior to statistical analysis according to Gill and Jones [35]. In order to assess the microbiological safety over the course of beef burgers’ shelf-life, the *Salmonella* spp. detection was performed according to ISO 6579–1: 2017 [36] at T0, T1, T2 and T3, in compliance with Regulation (EC) No. 2073/2005 [37].

Corresponding with the end of the manufacturing process (T0), the enumeration of *E. coli* was performed according to ISO 16649 [38] on all experimental groups as a process hygiene criterion of Regulation (EC) No. 2073/2005 [37].

### 2.7. Statistical Analysis

Data were analyzed using the GLM procedure of SAS [39]. An ANOVA model was used with sample (C, A, AP, P) and time (T0, T1, T2, T3) as the fixed factors. The replicate effect was found not significant and removed from the model. The differences in the means were detected using the Tukey’s test and considered significant when *p* < 0.05. The effects of formulation on the growth of the target micro-organisms were evaluated with the DMFit tool of the free predictive microbiology software Combase (https://www.combase.cc/index.php/en/DMFit, accessed on 20 June 2022), allowing for the definition of growth parameters such as lag phase duration (λ, 1/h) and maximum growth rate (µmax, 1/h) by means of the Baranyi and Roberts model [40]. The fitted results were analyzed by one-way ANOVA (with the sample as a fixed variable) and Tukey’s test (*p* < 0.05).

## 3. Results and Discussion

A higher antioxidant activity was found in the PE in comparison with the CM containing ascorbic acid (554.42 ± 3.38 and 1149.02 ± 13.69 µg TE/100 g in CM and PE, respectively). The high antioxidant activity recorded in the PE is in agreement with a previous studies that reported the powerful antioxidant activity of olive phenolic compounds [22,41,42,43,44]. The results for the lipid oxidation and antioxidant activity of minced beef meat indicated by TBARs and ORAC_FL_ values are reported in Table 2.

Concerning lipid oxidation, increased TBARs values were detected during storage for all experimental formulations, and, among groups, differences were reported starting from 2 days with higher values for C samples. The addition of 700 mg/kg PE in meat (P group) guaranteed the lowest level of TBAR_S_ in the second part of storage (days 5–7) with a reduction of 62% of lipidic oxidation on day 7 for P compared to C samples. Similar results, albeit with a lower magnitude, have been reported by Martínez-Zamora et al. [43], demonstrating how the incorporation of synthetic hydroxytyrosol reduced the oxidation of lamb patties by 35% with respect to the control sample at the end of shelf-life. The hamburger formulation and storage time significantly affected the lipid oxidation, and the interaction between these two factors was also significant (Table 2). A PE-concentration-dependent effect preserving lipid oxidation was also observed by other studies in the literature both in raw and grilled beef burger [20].

The ORAC_FL_ assay revealed differences in the antioxidant activity of four experimental groups, with the highest mean values recorded in the P group (Table 2). A reduction in the antioxidant activity during storage was recorded in A, AP and P. This reduction, as found in a previous study, is due mainly to the oxidative degradation phenomena of phenols or antioxidant molecule that occur during storage [20,44]. Despite the degradation of phenols, the higher level of integration in beef meat (P group) ensures considerable antioxidant activity until the end of storage time. It is well-known that phenols can act as hydrogen donors and compounds linked with a o-dihydroxyl functionality possess a high antioxidant activity, due to the formation of intramolecular hydrogen bonds observed during the reaction with free radicals [22]. Among these, the highest antioxidant activity was attributed to 3,4-DHPEA (hydroxytyrosol) and secoiridoid derivatives such as 3,4-DHPEA-EDA (oleacein) [22]. In particular, hydroxytyrosol’s strong antioxidant potential is strictly related to its chemical structure: a phenol ring formed by a catechol group and three hydroxyl groups [43]. The combination of these functional groups could represent the main explanation for its preservative action in products of animal origin, as previously demonstrated in the literature [45,46].

The preliminary evaluation of the antimicrobial activity of the CM and PE performed through the agar well diffusion technique revealed that the PE possessed greater in vitro antimicrobial activity compared to the CM. Indeed, after incubation, the inhibition halos were measured for each strain, and the PE showed halos of 16, 13 and 11 mm for *P. fluorescens*, 14, 11 and 9 mm for *S. aureus*, and 9, 7 and 0 mm for *E. coli* for 750, 375 and 187 mgPE/mL, respectively. The assay’s results suggest that the CM had no effect on microbial growth as the inhibition halos were absent for all the concentrations and micro-organisms tested. 

Concerning the microbial analysis of beef burger, the *Salmonella* spp. detection showed that the pathogen was absent during the entire duration of products’ shelf-life in all the experimental groups, complying with the food safety criterion of EU Regulation [37]. Similarly, the process hygiene criterion of *E. coli* in meat preparations was fully respected as the microbial count at T0 was below 2 Log CFU/g for all experimental groups [37]. This evidence confirms both the satisfactory safety and the hygiene levels of the beef burger production process. 

The results of microflora evolution during storage for refrigerated beef burgers are depicted in Table 3. As shown, a significant (*p* < 0.001) increase was observed for all microbial populations and for all experimental groups studied as storage time elapsed. Immediately after production (T0), higher microbial populations were recorded for the TVC and LAB count followed by *Pseudomonas* spp. The initial (T0) TVC in all studied groups was approximately 4.4 Log CFU/g, which can be considered a characteristic value for minced meat products after manufacturing [47]. Indeed, this result is in agreement with the levels reported in the available literature for similar minced beef meat products [48,49], albeit other studies have found higher values [50] or lower ones [51]. It has been reported that a possible explanation for this relatively high initial TVC contamination in beef burgers may be attributed to the mincing process, which contributes to the total viable counts, likely as a consequence of the disruption of muscle structure, making nutrients easily available to micro-organisms [49]. However, the low initial value of *Enterobacteriaceae* counts (average value 1.33 Log CFU/g) confirms the optimal initial microbiological quality attributable to the good physiological status of the animal at slaughter and to proper postmortem meat acidification as well as to the good hygienic conditions during slaughter, handling and production processes [47,52].

As above mentioned, following TVC, LAB and *Pseudomonas* spp. where the two microbial population with the highest initial value (T0), with average levels among experimental groups of 4.09 and 4.05 Log CFU/g, respectively. Similar counts were recorded for analogous products by Zamuz et al. [47] and Andres et al. [53], while slightly higher values were recorded by Parafati et al. [54] and Marrone et al. [55] for *Pseudomonas* in minced beef meat products.

At the end of the storage period (day 7), significant (*p* < 0.001) differences between experimental groups were observed for TVC, *Pseudomonas* and *Enterobatteriaceae* (Table 3). Specifically, the lowest value for this microbial population corresponded to those burgers formulated with the highest amount of PE (P group), suggesting that the bioactive molecules contained in the extract affected this microbial population by limiting its growth, as preliminary suggested by an in vitro assay.

The parameters characterizing the growth curves of targeted microbial populations in the four experimental groups were obtained by modeling growth data by means of the Baranyi equation [40] and are summarized in Table 4.

Regarding the TVC, the addition of polyphenols in beef burger resulted in an extended lag phase (λ) in P samples in comparison with C and A and a reduction in the maximum growth rate (μmax) in AP compared with all other groups and the final value for P. For *Staphylococcus* spp., the experimental treatment affected the microbial growth by extending the λ in P samples in comparison with C and A and by reducing the μmax in P compared with all other groups and in AP compared to C. The final value was also affected by a significant reduction in the two experimentally manufactured burger groups (AP, P). Considering *Pseudomonas* spp., the P group recorded a longer λ compared to C and A samples while AP was not statistically different from the other experimental groups; concerning μmax, both AP and P showed lower values compared to C and A. For this microbial population, the final value was significantly reduced in the P group.

Neither λ nor the final value of the LAB population were different among groups, while the μmax was slightly higher in AP and P compared to C and A, agreeing with previous results from Servili et al. [56], who reported that olive by-product polyphenols did not affect LAB growth in fortified foodstuff. For *Enterobacteriaceae*, data show a reduction in the final value in the AP and P, while no significant differences were highlighted for λ and μmax; however, an increasing trend was recorded for λ and a decreasing one was noted for μmax (*p* = 0.06 and *p* = 0.056, respectively, data not shown).

In agreement with what is reported above, Mexis et al. [57] noticed a reduced growth rate in TVC, LAB and pseudomonads in ground chicken meat with the addition of Citrus spp. extracts. In Mortadella meat products with citrus fiber, thyme and rosemary, essential oil lowered the growth rate of the TVC during storage [58]. Roila et al. [25] reported that the addition of olive oil by-product polyphenols in Fior di Latte cheese brine resulted in an extended λ for *P. fluorescens* and *Enterobacteriaceae* and in a reduced μmax for *P. fluorescens*.

As shown, the result of growth data modeling appears to be related to the influence exerted by the experimental addition of olive-mill-wastewater-derived polyphenols on extending the λ and reducing the μmax and the final value. Analogous conclusions have been previously reported for similar compounds and for other preservation methods, albeit a systematic comparison is difficult as the characteristics of microbial growth curves can be affected by differences in food matrices, storage condition and duration [25,59].

Concerning the antimicrobial activity, it has been demonstrated that the dialdehydic structure of olive phenols exerts an antimicrobial effect by strongly interacting with amino acids, proteins and membrane molecules, promoting membrane permeabilization and bacterial cell lysis [60]. Indeed, studies have shown that tyrosol inhibits the activity of cyclooxygenase enzymes and hydroxytyrosol has a protein-denaturing ability [60]. Other studies report that many polyphenolic compounds are potent iron scavengers, and the lack of iron affects the growth of certain pathogenic bacteria by a reduction in the ribonucleotide precursor of DNA [61]. Besides the molecular mechanisms, the microbial growth inhibition exerted by this phenolic compound is strongly related to its chemical structure; as a consequence, the key factor determining the antibacterial activity of the phenolic extracts is their phenolic profile [62]. 

## 4. Conclusions

Consumers’ interest in meat products formulated with natural preservatives has motivated researchers to evaluate the effectiveness and applicability of naturally occurring compounds with antioxidant and antimicrobial purposes. This study has proved that olive mill wastewater extracts are characterized by a phenolic profile and are able to significantly improve the oxidative and microbial stability of beef burger during cold storage.

Therefore, it was concluded that olive mill wastewater extracts could be successfully added to raw ground beef meat to act as natural antioxidants and antimicrobials with added health, environmental and economic benefits as well as increased consumer appeal.

## Figures and Tables

**Table 1:** Composition of spray-dried crude phenolic extract (PE).

Crude PE (mg/g)	
3,4-DHPEA *	9.2 ± 0.2
*p*-HPEA	4.3 ± 0.0
Verbascoside	5.9 ± 0.2
3,4-DHPEA-EDA	8.1 ± 0.2
*Sum of phenols*	*27.6 ± 0.3*
*Purity*	*2.7%*

* Results are the mean of two independent analytical determinations ± standard deviation. 3,4-DHPEA = hydroxytyrosol, *p*-HPEA = tyrosol, 3,4-DHPEA-EDA = oleacein.

**Table 2:** Lipid oxidation (TBAR_S_) and antioxidant capacity (ORAC_FL_) in beef burger during storage.

		Days of Storage				SEM	*p*-Value		
		0	2	5	7		T	S	TXS
TBARS (mg MDA/kg)	C	0.13 ^a^	0.34 ^bW^	0.47 ^cW^	0.63 ^dW^				
	A	0.14 ^a^	0.20 ^bX^	0.25 ^cX^	0.30 ^dX^	0.012	<0.001	<0.001	<0.001
	AP	0.14 ^a^	0.17 ^abXZ^	0.19 ^bZ^	0.29 ^cX^				
	P	0.15 ^a^	0.16 ^aZ^	0.18 ^aZ^	0.24 ^bZ^				
ORAC_FL_ (µg TE /100 g)	C	24.44 ^W^	24.43 ^W^	24.44 ^W^	24.41 ^W^				
	A	34.19 ^aX^	32.11 ^bX^	27.08 ^cX^	25.59 ^dW^	0.475	<0.001	<0.001	<0.001
	AP	38.87 ^aY^	38.18 ^aY^	36.17 ^bY^	35.89 ^bX^				
	P	44.45 ^aZ^	43.89 ^aZ^	40.01 ^cZ^	38.73 ^bY^				

C = control group; A = basic recipe with addition of 10 g/kg commercial antioxidant; AP = basic recipe with addition of 5 g/kg commercial antioxidant and 350 mg/kg phenolic extract; P = basic recipe with addition of 700 mg/kg phenolic extract. Different letters in the same row (a, b, c, d) indicate differences between mean values during sampling times (*p* ≤ 0.001); different letters in the same column (W, X, Y, Z) indicate differences between mean values for different experimental groups (*p* ≤ 0.001). SEM, standard error of the mean. T = time; S = sample.

**Table 3:** Microbial quality (Log CFU/g) of the four formulations of beef burger stored at 4 °C under aerobic conditions for 7 days.

		Days of Storage				SEM	*p*-Value		
		0	2	5	7		T	S	TXS
TVC	C	4.29 ^a^	5.50 ^b^	6.85 ^cW^	7.41 ^dW^				
	A	4.37 ^a^	5.48 ^b^	6.82 ^cW^	7.38 ^dW^	0.124	<0.001	<0.001	0.002
	AP	4.61 ^a^	5.31 ^b^	6.36 ^cX^	6.98 ^dWX^				
	P	4.48 ^a^	5.11 ^b^	6.24 ^cX^	6.52 ^cX^				
*Staphylococcus* spp.	C	1.46 ^a^	2.07 ^b^	2.78 ^cW^	2.89 ^c^				
	A	1.28 ^a^	1.90 ^b^	2.67 ^cW^	2.87 ^c^	0.159	<0.001	0.005	0.671
	AP	1.32 ^a^	1.78 ^a^	2.47 ^bWX^	2.67 ^b^				
	P	1.38 ^a^	1.67 ^ab^	2.05 ^bc^^X^	2.49 ^c^				
*Pseudomonas* spp.	C	4.02 ^a^	5.18 ^b^	6.67 ^cW^	7.05 ^dW^				
	A	4.11 ^a^	5.23 ^b^	6.75 ^cW^	7.20 ^dW^	0.199	<0.001	<0.001	0.163
	AP	4.09 ^a^	4.74 ^b^	5.96 ^cX^	6.91 ^dWX^				
	P	3.98 ^a^	4.47 ^a^	5.63 ^bX^	6.11 ^bX^				
LAB	C	4.15 ^a^	4.46 ^ab^	4.83 ^b^	4.96 ^b^				
	A	4.20 ^a^	4.48 ^ab^	4.79 ^b^	4.88 ^b^	0.142	<0.001	0.508	0.999
	AP	4.04 ^a^	4.39 ^ab^	4.82 ^b^	4.94 ^c^				
	P	3.98 ^a^	4.29 ^ab^	4.75 ^b^	4.83 ^c^				
*Enterobacteriaceae*	C	1.38 ^a^	1.80 ^ab^	2.58 ^c^	3.60 ^dW^				
	A	1.30 ^a^	1.66 ^a^	2.53 ^b^	3.58 ^cW^	0.182	<0.001	<0.001	0.102
	AP	1.24 ^a^	1.46 ^a^	2.27 ^b^	3.17 ^cW^				
	P	1.28 ^a^	1.36 ^a^	2.10 ^b^	2.36 ^bX^				

C = control group; A = basic recipe with addition of 10 g/kg commercial antioxidant; AP = basic recipe with addition of 5 g/kg commercial antioxidant and 350 mg/kg phenolic extract; P = basic recipe with addition of 700 mg/kg phenolic extract. Different letters in the same row (a, b, c, d) indicate differences between mean values during sampling times (*p* ≤ 0.001); different letters in the same column (W, X, Y, Z) indicate differences between mean values for different experimental groups (*p* ≤ 0.001). SEM, standard error of the mean. T = time; S = sample.

**Table 4:** Output parameters estimated by the DMFit program for each microbial population in the four formulations of beef burgers.

Micro-Organism and Parameters	C	A	AP	P
TVC				
λ	8.97 ± 3.45 ^a^	6.12 ± 3.45 ^a^	15.84 ± 8.20 ^ab^	23.27 ± 11.74 ^c^
µmax	0.0286 ± 0.0014 ^b^	0.0286 ± 0.0013 ^b^	0.0218 ± 0.0024 ^a^	0.0248 ± 0.0048 ^b^
Final value	7.44 ± 0.03 ^b^	7.44 ± 0.03 ^b^	7.11 ± 0.07 ^b^	6.62 ± 0.10 ^a^
*R^2^*	*0.999*	*0.999*	*0.995*	*0.98*
*SE of Fit*	*0.041*	*0.0397*	*0.080*	*0.139*
*Staphylococcus* spp.				
λ	10.78 ± 5.35 ^a^	11.86 ± 3.64 ^a^	17.95 ± 3.82 ^ab^	20.84 ± 6.03 ^b^
µmax	0.0163 ± 0.0015 ^bc^	0.0171 ± 0.0010 ^c^	0.0151 ± 0.0009 ^b^	0.0092 ± 0.0001 ^a^
Final value	2.92 ± 0.02 ^b^	2.89 ± 0.02 ^b^	2.69 ± 0.02 ^a^	2.66 ± 0.00 ^a^
*R^2^*	*0.997*	*0.999*	*0.998*	*0.998*
*SE of Fit*	*0.0346*	*0.0262*	*0.0263*	*0.065*
*Pseudomonas* spp.				
λ	12.33 ± 9.84 ^a^	14.33 ± 8.32 ^a^	20.38 ± 9.25 ^ab^	29.73 ± 2.07 ^b^
µmax	0.0324 ± 0.004 ^b^	0.033 ± 0.004 ^b^	0.024 ± 0.003 ^a^	0.025 ± 0.000 ^a^
Final value	7.16 ± 0.10 ^b^	7.30 ± 0.09 ^b^	7.07 ± 0.11 ^b^	6.10 ± 0.02 ^a^
*R^2^*	*0.99*	*0.993*	*0.998*	*0.999*
*SE of Fit*	*0.136*	*0.12*	*0.11*	*0.025*
LAB				
λ	10.66 ± 8.58	9.86 ± 4.44	11.33 ± 3.19	17.03 ± 4.67
µmax	0.0083 ± 0.0010 ^a^	0.0071 ± 0.0000 ^a^	0.0095 ± 0.0040 ^b^	0.0102 ± 0.0002 ^b^
Final value	4.94 ± 0.02	4.89 ± 0.09	4.95 ± 0.08	4.83 ± 0.12
*R^2^*	*0.993*	*0.998*	*0.999*	*0.997*
*SE of Fit*	*0.0294*	*0.013*	*0.0128*	*0.0209*
*Enterobacteriaceae*				
λ	34.38 ± 10.82	38.59 ± 6.21	44.40 ± 2.55	49.036 ± 10.74
µmax	0.0205 ± 0.0026	0.022 ± 0.0015	0.0201 ± 0.0006	0.0176 ± 0.0035
Final value	3.80 ± 0.12 ^c^	3.88 ± 0.07 ^c^	3.38 ± 0.025 ^b^	2.41 ± 0.05 ^a^
*R^2^*	*0.989*	*0.996*	*0.999*	*0.987*
*SE of Fit*	*0.112*	*0.0672*	*0.0238*	*0.063*

λ = lag phase (h); µmax = maximum growth rate (Log/CFU/g/h); final value (Log/CFU/g); SE = standard error of fitting; R^2^ = adjusted R-square statistics of the fitting. C = control group; A= basic recipe with addition of 10 g/kg commercial antioxidant; AP = basic recipe with addition of 5 g/kg commercial antioxidant and 350 mg/kg phenolic extract; P = basic recipe with addition of 700 mg/kg phenolic extract. Different letters in the same row (a, b, c) indicate differences between mean values for different experimental groups (*p* ≤ 0.001).

## Data Availability

The datasets generated for this study are available on request from the corresponding author.
